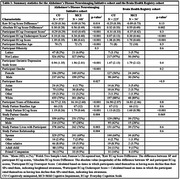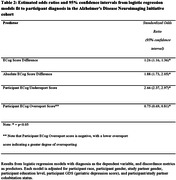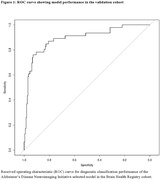# Discordance between self‐ and study partner‐reported cognitive decline is associated with MCI diagnosis

**DOI:** 10.1002/alz.087051

**Published:** 2025-01-03

**Authors:** Anna Aaronson, Adam Diaz, Miriam T. Ashford, Chengshi Jin, Rachana Tank, Melanie J. Miller, Jaemyeong Kang, Manchumad Manjavong, Bernard Landavazo, Joseph Eichenbaum, Diana Truran‐Sacrey, Monica R. Camacho, Juliet Fockler, Derek Flenniken, Scott R. Mackin, Michael S. W. Weiner, Rachel L. Nosheny, the Alzheimer’s Disease Neuroimaging Initiative

**Affiliations:** ^1^ University of California, San Francisco, San Francisco, CA USA; ^2^ San Francisco Veterans Affairs Medical Center, San Francisco, CA USA; ^3^ Northern California Institute for Research and Education (NCIRE), San Francisco, CA USA; ^4^ Veterans Affairs Medical Center, San Francisco, CA USA; ^5^ Dementia Research Center UCL Institute of Neurology University College London, London United Kingdom; ^6^ Gil Medical Center, Gachon University College of Medicine, Incheon Korea, Republic of (South); ^7^ Khon Kaen University, Khon Kaen Thailand; ^8^ VA Advanced Imaging Research Center, San Francisco Veterans Affairs Medical Center, San Francisco, CA USA

## Abstract

**Background:**

In Alzheimer’s disease research, subjective report of cognitive and functional decline from participant‐study partner (SP) dyads is an efficient method of assessing cognitive impairment and risk of clinical progression. The extent to which discordance (disagreement) between self‐ and SP–report is associated with diagnosis of cognitive impairment is not known.

**Method:**

We tested the hypothesis that discordance between baseline self‐ and SP‐report Everyday Cognition Scale (ECog) scores was associated with greater probability of mild cognitive impairment (MCI) diagnosis. Dyads enrolled in the Alzheimer’s Disease Neuroimaging Initiative (ADNI) and the University of California, San Francisco Brain Health Registry (BHR), an online longitudinal aging‐related research registry, completed an online adaptation of the 39‐item ECog to assess subjective change across six cognitive domains.

We derived four metrics of discordance between participant and SP ECog scores (dyadic discordance): Raw Score Difference, Absolute Score Difference, Overreport Score, and Underreport Score (Table 1). In the ADNI cohort, we fit a logistic regression model for each of the discordance metrics to independently evaluate their association with MCI diagnosis, after adjusting for dyad relationship and sociodemographic factors. Then, to further evaluate the predictive utility of these measures, we carried out a model selection procedure using cross‐sectional data collected from ADNI dyads (N = 921; Table 1). Finally, we externally validated the model in a BHR cohort with clinically confirmed diagnoses (N = 279; Table 1).

**Result:**

Higher Raw and Absolute Score Difference, greater Underreport scores, and lower Overreport scores were associated with greater probability of MCI in the ADNI cohort (Table 2). The model selection procedure identified a number of highly predictive variables, which were then included in a model that was externally validated in the BHR cohort. This model distinguished diagnostic groups in the BHR cohort with AUC = 0.892, Sensitivity = 0.61, Specificity = 0.95 (Figure 1) based on a restricted cubic spline regression model.

**Conclusion:**

Results indicate that ECog score discordance is associated with MCI diagnosis. The selected model showcased good predictive performance in the validation cohort, and highlights the potential utility of subjective dyadic discordance metrics to help identify older adults with MCI in diverse settings.